# Surgical treatment of a unilateral congenital cataract with persistent hyperplastic primary vitreous using double optic capture with a 20 year follow-up and review of the literature

**DOI:** 10.1016/j.ajoc.2025.102341

**Published:** 2025-04-22

**Authors:** Brian M. DeBroff

**Affiliations:** Department of Ophthalmology and Visual Science, Yale University School of Medicine, New Haven, CT, USA

**Keywords:** Optic capture, Double optic capture, Congenital cataract, Persistent hyperplastic primary vitreous, PHPV, Posterior capsulorhexis

## Abstract

**Purpose:**

To describe the long term follow-up of surgical intervention with the technique of Double Optic Capture to surgically treat a unilateral congenital cataract with hyperplastic primary vitreous and the ability to perform intraocular lens exchange of a double captured intraocular lens 17 years after the initial procedure.

**Observations:**

An eight week old with unilateral cataract with persistent hyperplastic primary vitreous of the left eye had cataract surgery which included anterior capsulorhexis, a primary posterior capsulorhexis, anterior vitrectomy and placement of a posterior chamber intraocular lens implantation through both capsulotomy openings (double optic capture). Fourteen years later, patient was best corrected to 20/20 vision with a refraction of −14.75–2.00 x 100. Due to difficulties with anisometropia, the patient underwent successful intraocular lens exchange. The intraocular lens was able to be successfully removed from its double captured position and a new implant was successfully re-captured through the fused capsule. Two years follow-up after IOL exchange, the implant was well centered and clear of any secondary membranes or scar tissue with a best corrected visual acuity of 20/20-2.

**Conclusions:**

This represents one of the longest reported follow-up with not only unilateral cataract with persistent hyperplastic primary vitreous, but also of the technique of double optic capture and successful intraocular lens exchange following the technique of double optic capture.

## 1 Introduction

Persistent hyperplastic primary vitreous (PHPV) can be associated with cataract, glaucoma, shallow anterior chambers, retrolental membranes, vitreous stalk, preretinal membranes, retinal folds, and optic hypoplasia.^1^, ^2^, ^3^ Most studies reported surgical results of infants undergoing lensectomy with or without vitrectomy typically have limited follow-up (less than 76 months).^1^, ^2^, ^3^ Due to the high rate of amblyopia often associated with unilateral congenital cataracts and the higher associated co-morbidities associated with PHPV, few series have reported more than a limited number of patients achieving 20/40 or better vision.^3^^,^^4^ Even with early surgery, as is often required in cases of PHPV with unilateral cataract, this condition has a high rate of need for secondary surgical procedure including capsulotomies.^5^ Primary intraocular lens (IOL) implantation is recommended as postoperative complication rates are increased with aphakia in PHPV infants.^6^ For infants presenting with unilateral cataracts and PHPV, surgical management needs to address removal of the lenticular opacity, removal of any retrolental membranes and vitreous stalks, while maintaining IOL centration and prevention of secondary membranes.

Double optic capture (DOC) was first described in 2008 as a method to reduce visual axis opacification while ensuring IOL centration in pediatric cataract surgery.^7^ DeBroff and Nihalani presented 16 eyes with a mean follow-up of 1.7 years that maintained IOL centration, had minimal anterior chamber reaction, and no evidence of visual axis opacification.^7^ The technique involves performing a continuous curvilinear capsulorhexis of the anterior capsule, removal of the cataract, performing a posterior capsulotomy opening (either using a vitrectomy handpiece to match the size of the anterior capsulorhexis or performing a primary posterior capsulorhexis), anterior vitrectomy if needed, placement of IOL haptics in the ciliary sulcus, followed by capture of the IOL optic through both the anterior and posterior capsular openings.^7^, ^8^, ^9^, ^10^ Ideally the posterior capsular opening should be at least 1 mm smaller than the IOL optic to allow for optimal capture. This technique allows for fusion of the anterior and posterior leaflets of the capsular bag for 360°, effectively sealing the capsular bag. This capsular fusion reduces the possibility of lens epithelial cell migration and thus reduces the incidence of secondary membrane formation while locking in the IOL position and preventing decentration. Finally, due to the secure and stable fixation of the IOL, uveal haptic rub is minimized and low anterior chamber inflammation is observed postoperatively. Onol et al., in 2000, described using DOC technique in combination with pars plana lensectomy in pediatric cataracts^11^ and subsequently published a series with long term follow-up demonstrating IOL stability and clarity of the visual axis in 16 eyes.^12^ Other surgeons have described excellent results utilizing the double optic capture technique as a preferred technique to maintain IOL centration and prevent secondary membrane formation.^13^ Herein, this case report describes a 20 year follow-up of a patient presenting with a unilateral cataract and PHPV, who not only underwent the double optic capture technique, but also successfully had IOL exchange 17 years after the initial surgery and a final best corrected visual acuity of 20/20-2.

## 2 Case report

A one month old female presented to the Yale Eye Center with absence of red pupillary reflex of her left eye and was found to have a dense posterior polar cataract and persistent hyaloid vasculature. She was born one month prior to presentation at 39 weeks gestation, weighing 8.0 lbs. On presentation, general examination was unremarkable with symmetrical corneal reflexes and no relative afferent pupillary defect. Dilated fundus examination revealed left leukocoria due to a posterior polar cataract associated with a fibrovascular sheath attachment from the posterior capsule, extending posteriorly into the vitreous consistent with persistent hyperplastic primary vitreous. The posterior polar cataract blocked the view of the optic disc and macula; the peripheral retina examination was unremarkable.

**Fig. 1:**
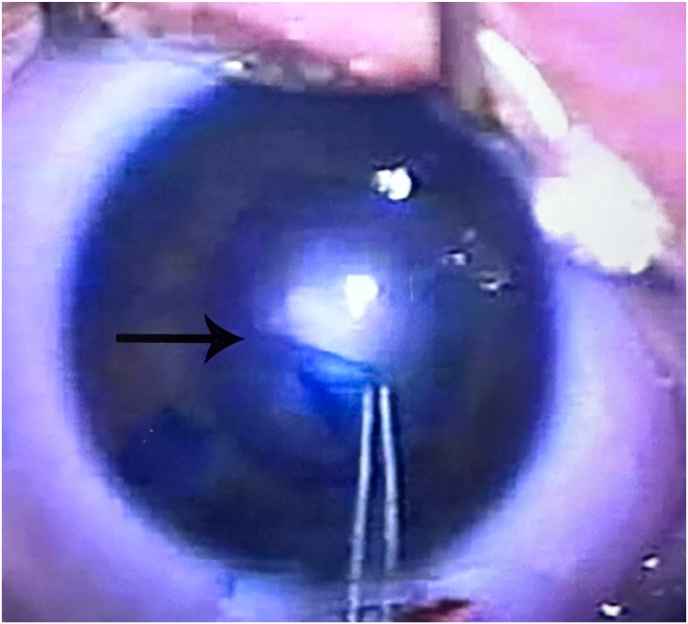
The final steps of the anterior continuous curvilinear capsulorhexis and the appearance of the extent of the posterior polar cataract during the primary surgical procedure Left eye (arrow shows leading edge of the anterior continuous curvilinear capsulorhexis).

**Fig. 2:**
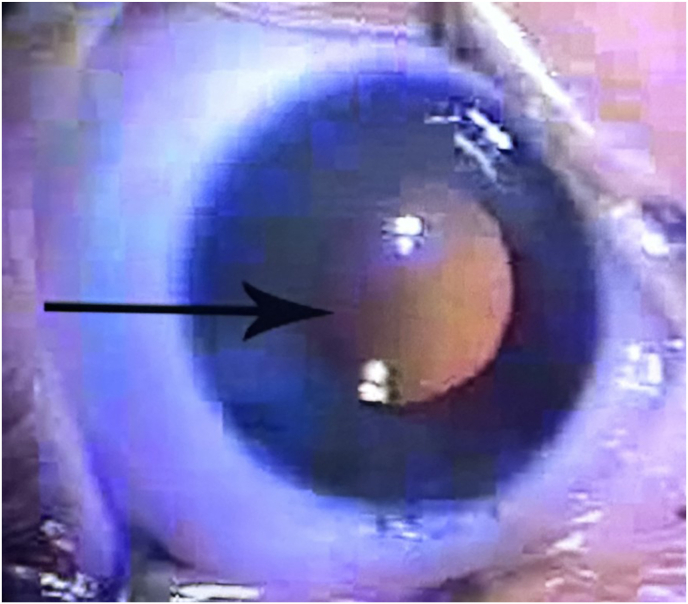
Appearance of the IOL Left eye in its double captured position at the conclusion of primary surgical procedure (arrow shows double captured IOL).

After informed consent was obtained from the patient's mother and father, the patient underwent examination under general anesthesia and surgical repair at eight weeks of age. Intraocular pressure was found to be 9 OD and 21 OS, corneal diameters were 10 mm horizontally OU. Keratometry readings were 44.75 x 46.50 OD and 46.94 x 49.19 OS. Axial length OD was 17.85 and OS was 18.33. Surgical removal of the cataract OS included an anterior continuous capsulorhexis and lens aspiration using the irrigation/aspiration handpiece. Fig. 1 shows the final steps of the anterior continuous capsulorhexis and the extent of the posterior polar cataract. A dense posterior calcific plaque was noted upon removal of the nuclear and cortical material. A primary continuous curvilinear posterior capsulorhexis (PCCC) was performed which encompassed the posterior plaque. The plaque with attached vascular stalk was removed and sent to pathology without evidence of bleeding. Later pathological examination by Yale Ophthalmic Pathology confirmed fibrovascular connective tissue consistent with PHPV. A limited anterior vitrectomy was performed to remove additional fibrovascular material with no evidence of bleeding. Yale retina service was present in the OR on standby in case of need for cautery of vascular stalks or more extensive vitrectomy and they performed an exam under anesthesia which demonstrated no tractional bands, no bleeding, and a only a small stalk from the surface of the optic nerve (Bergmeister Papilla). A posterior chamber IOL: AcrySof MA60AC, power +30.0 diopters (Alcon Inc, Fort Worth TX) was placed with the haptics in the ciliary sulcus and then the IOL optic was captured through both the anterior and posterior capsule openings to achieve Double Optic Capture (DOC). Fig. 2 shows appearance of the IOL in its double captured position at the conclusion of surgery.

The patient was managed post-operatively with the Yale anterior segment service, Yale pediatric ophthalmology service, Yale Retina & Vitreous service, and Yale Glaucoma service (history of borderline IOP/glaucoma suspect requiring no pharmacological or surgical intervention). The patient's initial hyperopia was treated and patching was strictly adhered to. She maintained excellent visual acuity with progressive myopia. By age 14, she presented with difficulties wearing her contact lens and anisometropia with a refraction OS of -14.75–2.00 x100 (20/20 vision OS, best corrected). The posterior chamber IOL remained in the captured location with a completely clear visual axis and no evidence of secondary membranes or fibrosis.

**Fig. 3:**
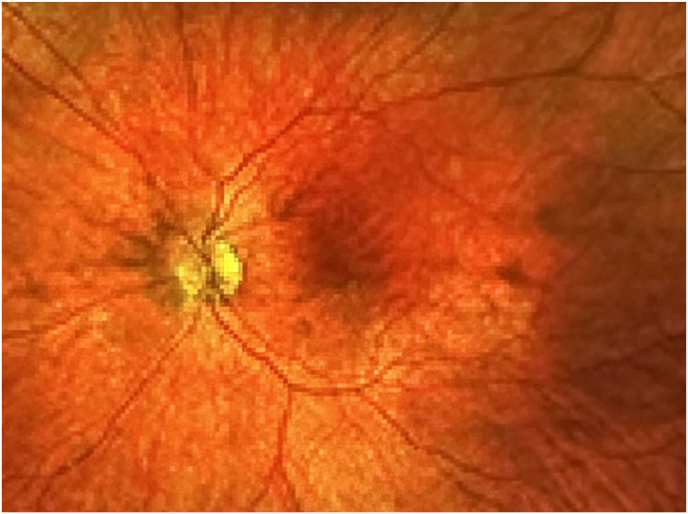
Fundus Left Eye (after IOL exchange) demonstrating resolution of Bergmeister papilla and minimal cupping of the optic nerve.

**Fig. 4:**
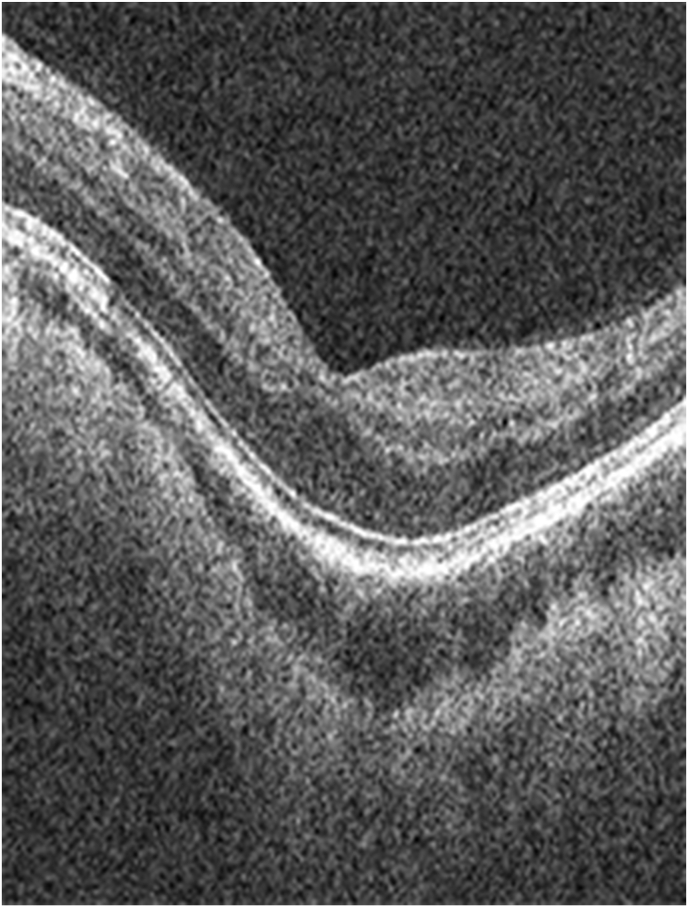
Macular OCT (after IOL exchange) demonstrating normal foveal contour and no evidence of cystoid macular edema.

At age 17, after informed consent was reviewed and signed by the patient and her parents, she underwent an IOL exchange under general anesthesia with ORA system intraoperative aberrometer (Alcon Inc, Fort Worth Tx) due to anisometropia and contact lens intolerance. The implant optic was moved anteriorly from its double capture position into the anterior chamber without difficulty, capsular rent, or zonular dehiscence. The IOL was bisected into 2 pieces using an IOL cutter and removed through the main incision. A limited anterior vitrectomy was performed to create a round approximately 5 mm diameter round fused capsular opening. A three-piece IPURE posterior chamber IOL (P3PC), power 13.0 diopter (BVI Medical, Waltham MA) was placed with the haptics in the sulcus and the optic was recaptured through the fused capsular opening to achieve stable capture. Post-operative course was unremarkable and examination two years after IOL exchange revealed the IOL to be in excellent position, no secondary membranes, and a manifest refraction of −2.00 – 1.00 x 95 with best-corrected vision of 20/20-2. Fundus photography of the left eye revealed complete regression of the Bergmeister papilla OS (Fig. 3) and no cystoid macular edema on macular optical coherence tomography (Fig. 4).

## 3 Discussion

Congenital unilateral cataracts in infants, even when treated with early surgical intervention, are typically associated with poor visual outcomes due to amblyopia, secondary membranes, and often the need for repeated surgical procedures.^14^ Cheng et al. reported results of a series that 60 % achieved 20/200 or less vision and only 20 % achieving 20/40 or better.^14^ The results of congenital unilateral cataracts associated with PHPV typically fare even worse with Hunt et al. reporting only 18 % of their series achieving better than 20/200 vision.^1^ Very few studies have data past seven years of age for unilateral cataracts with PHPV.^4^

This case report highlights significant achievements in treating an infant with unilateral cataract with PHPV, namely: 1) a patient with 20 year follow-up, 2) early IOL implantation using the double optic capture technique, 3) documentation of successful IOL exchange of a double-captured IOL after 17 years, and 4) the achievement of 20/20 vision in a unilateral congenital PHPV cataract with histopathologic confirmation of PHPV. Critical measures to achieve the results in this case presented include, early surgical intervention, a surgical technique that involves removal of the posterior lenticular plaque and vitreous stalks, placement of an IOL that will remain centered, and a technique that minimizes secondary membranes and retro-lenticular membranes that are frequently associated with infantile and pediatric cataract surgery, and finally, meticulous amblyopia management throughout childhood. The double optic capture technique has been shown to be successful in performing pediatric IOL stability and preventing secondary membrane formation,^7^, ^8^, ^9^, ^10^, ^11^, ^12^, ^13^^,^^15^ and played an important factor in the successful outcome presented in this patient.

DeBroff and Gimbel in 2004 reported that DOC can be achieved by performing both an anterior capsulorhexis and posterior capsulorhexis and placing the IOL optic through both the anterior and posterior capsulorhexis openings.^15^ Because performing a posterior capsulorhexis can be technically challenging, especially to create a circular opening approximately 1 mm smaller than the IOL optic diameter in an elastic pediatric capsule, femtosecond laser may be a future option to perform not only the anterior capsular opening, but also the posterior capsular opening.^16^, ^17^, ^18^, ^19^, ^20^ For surgeons, either not trained or comfortable performing posterior continuous capsulorhexis, the technique of using an anterior vitrector handpiece to create a circular opening in the posterior capsulotomy smaller than the IOL optic has been shown to be successful and safe in enabling secure double optic capture.^7^ Surgeons, who are technically adept with consistently performing a posterior capsulorhexis of a diameter 1 mm or more smaller than the optic diameter, may consider the technique of posterior capsulorhexis with optic capture (PCOC), which involves in-the-bag IOL placement with optic capture through the posterior capsulorhexis.^21^^,^^22^ The downside to PCOC (as compared to double optic capture) is that the fusion of the anterior and posterior capsules are not complete 360° (discontinuous at the optic haptic juncture)^23^^,^^24^ and extension of a posterior capsulotomy precludes the placement of the haptics in the capsular bag. If an extension of the capsulotomy does occur during the procedure, a sulcus IOL can be placed with optic capture through either the intact anterior or posterior capsulotomy opening.^7^

Performing a posterior capsulorhexis, however, with either DOC or PCOC has the benefit of potential elimination of the need to perform an anterior vitrectomy at the time of surgery.^25^^,^^26^ Avoiding an anterior vitrectomy may be beneficial to reduce inflammatory sequalae and decrease the incidence of cystoid macular edema especially in adult patients.^27^ In cases where removal of posterior lenticular plaques or vascular stalk remnants are needed, such as in cases of PHPV, a vitrectomy is required. Dr. Howard Gimbel presented a case of DOC utilizing posterior capsulorhexis without anterior vitrectomy to successfully treat a congenital pediatric cataract associated with Down's Syndrome.^28^ In 2014, DeBroff reported that with DOC, even if the anterior capsulorhexis is larger than the IOL optic, the posterior capsulotomy size can be adjusted smaller than the IOL optic to enable effective and secure capture.^8^ Also, IOL optic centration has been shown to be maintained even in cases of eccentric anterior capsulorhexis.^29^

Adult patients have been demonstrated to also benefit from the technique of double optic capture. Campos, Ahmed, and Shah presented a case of intraocular lens exchange of a sulcus placed single piece acrylic IOL which was causing uveitis, glaucoma, and hyphema (UGH) syndrome. After removal of the single piece IOL, a three piece IOL was placed in the sulcus and the optic was captured through both the anterior and posterior capsulotomy openings.^30^^,^^31^ The demonstrated use of DOC to treat an inflammatory, acute uveitis condition confirms the ability of this technique to stabilize the IOL and prevent uveal chaffing even with placement of haptics within the ciliary sulcus. The authors state this technique (DOC) reduced IOL movement and ensured adequate centration.^30^

The techniques of DOC and PCOC have also been utilized in adults to prevent posterior capsular opacity following cataract surgery. Simply relying on sharp edge design of IOL's is limited and is associated with negative dysphotopsias.^23^ Nd:Yag (YAG) capsulotomy to treat visually significant posterior capsule opacity is not without risk, especially with regard to a higher incidence of retinal breaks or detachments.^32^ Both pediatric and adult pseudophakia patients with posterior capsule opacity may require Nd:YAG laser for visually significant posterior capsule opacity. For children under the age of 6 and uncooperative older children, the Nd:YAG laser has been modified to enable posterior capsulotomy on children in the supine position. A 1994, publication by Atkinson and Hiles demonstrated the effectiveness of yag laser in children 3 months of age and older.^33^ In addition, Kinori et al. reviewed a large series of children have Nd:YAG laser capsulotomy successfully performed in the operating room using the lateral decubitus position.^34^ A meta-analysis study to evaluate the impact of YAG laser capsulotomy on the incidence of pseudophakic retinal detachment (RD) showed a relative risk ratio of 1.57.^35^ Other complications of YAG capsulotomy include cystoid macular edema, elevated intraocular pressure, and damage to the intraocular lens.^36^ In addition, the cost of YAG capsulotomies is significant; in 2010, the Center for Medicare Services (CMS) spent 187 million dollars for YAG capsulotomies in the CMS population alone.^37^ Rupert Menapace in 2006 first described a technique of performing a primary posterior capsulorhexis after injecting ophthalmic viscosurgical device (OVD) under the posterior capsule opening to separate the underlying hyaloid surface from the posterior capsule.^38^ Arbisser in 2021 explains in detail the surgical method to perform hyaloid sparing PCCC using cohesive OVD to separate the capsule and anterior hyaloid with preservation of Berger's space.^39^

Menapace called this technique posterior optic buttonholing and reported no significant opacification within the visual axis in 500 consecutive patients.^38^ In 2008, he reported a series of 1000 consecutive cases of this technique of posterior capsulorhexis with buttonholing and reported no cases of retrolental after-cataract formation.^40^ This technique would eliminate the need for sharp edge IOL's, thus decreasing the amount of associated visual distortion.^40^^,^^41^

Arbisser in 2022 describes that the hyaloid sparing technique can also be used with DOC (IOL optic being captured through both the anterior and posterior capsulotomies).^39^ Arbisser describes this technique as hyaloid-sparing double capture with placement of the IOL optic into Berger's space.^39^^,^^42^ Dr. Thomas Oetting reported 12 cases from the University of Iowa for which his preferred method was this hyaloid sparing double optic capture resulting in IOL centration and no posterior capsular opacification.^13^

Many surgeons are more comfortable with the DOC technique due to the 360° fusion of the capsules and less concern about posterior dislocation of an IOL due to too large or eccentric PCCC, and less concern about a IOL haptic becoming misplaced beneath the posterior capsulorhexis opening during the insertion process. In addition, the DOC technique allows for simultaneous posterior capsulotomy and anterior vitrectomy, which in the case of PHPV is necessary to remove any retrolental membranes or vitreous stalks while preventing secondary membranes and allowing long term IOL centration. This report highlights the ability and success to perform IOL exchange with double optic capture many years (17 years in this case) after the original surgery, if required.

## 4 Summary

PHPV associated with a unilateral cataract typically results in very poor visual outcomes due to secondary membranes and amblyopia. In the case presented in this paper, early surgical intervention at 8 weeks of age, performing both an anterior and posterior capsulorhexis, performing an anterior vitrectomy, capturing the IOL optic to prevent secondary membranes, excellent patching and refraction correction resulted in 20/20 visual acuity after 17 years follow-up. Due to the patient's progressive myopia and anisometropia, IOL exchange was performed 17 years after the original surgical procedure. This procedure resulted in successful removal of the captured IOL without sacrificing or compromising capsular support, and enabling IOL exchange with re-capturing of the IOL optic through the fused capsule opening. At two years follow-up after the IOL exchange, the patient's best corrected visual acuity was 20/20-2 with excellent IOL centration and no secondary membranes or scar tissue, approximately 20 years after the original surgical procedure.

The technique of double optic capture has been shown to achieve IOL centration and near elimination of secondary cataract in infants, children, and even adults. It also offers the advantage of incorporating anterior vitrectomy (when desired) which is especially useful in cases of retro-lenticular membranes or vitreous vasculature remnants (commonly seen with PHPV). Finally this case demonstrates the ability to effectively perform IOL exchange with double optic capture after 17 years with preservation of the capsule and even re-capture of a secondary IOL. In cases of PHPV where there is a premium on early intervention, need for an anterior vitrectomy, a high rate of secondary cataract and membrane formation, and high rate of amblyopia, double optic capture offers a compelling choice. In addition, more recent studies and a multi-center and multi-national study is currently being conducted to evaluate its possibility in reducing or eliminating secondary cataract formation in patients of all age groups.

## Statement of informed consent

Consent to publish this case report has been obtained from the patient in writing.

## Ethical approval

This case report was conducted in accordance with the Declaration of Helsinki. The collection and evaluation of all protected health information was performed in a Health Insurance Portability and Accountability Act (HIPAA)-compliant manner.

## Disclosures

No funding or grant support.

## Data statement

Data was generated at Yale University School of Medicine and Yale New Haven Health. Derived data supporting the finding of this study are available from the corresponding author (BMD) on request.

## Declaration of competing interest

The authors declare that they have no known competing financial interests or personal relationships that could have appeared to influence the work reported in this paper.
